# Impact of through‐slice gradient optimization for dynamic slice‐wise shimming in the cervico‐thoracic spinal cord

**DOI:** 10.1002/mrm.30543

**Published:** 2025-05-01

**Authors:** Arnaud Breheret, Alexandre D'Astous, Yixin Ma, Jason P. Stockmann, Julien Cohen‐Adad

**Affiliations:** ^1^ NeuroPoly Lab, Institute of Biomedical Engineering Polytechnique Montréal Quebec Canada; ^2^ Centre de Recherche du CHU Sainte‐Justine Université de Montréal Quebec Canada; ^3^ Athinoula A. Martinos Center for Biomedical Imaging, Department of Radiology Massachusetts General Hospital Charlestown Massachusetts USA; ^4^ Athinoula A. Martinos Center for Biomedical Imaging Massachusetts General Hospital Charlestown Massachusetts USA; ^5^ Harvard Medical School Boston Massachusetts USA; ^6^ Functional Neuroimaging Unit, CRIUGM Université de Montréal Quebec Canada; ^7^ Mila–Quebec AI Institute Quebec Canada

**Keywords:** AC/DC, dynamic slice‐wise B_0_ shimming, fMRI, multicoil, signal recovery, spinal cord

## Abstract

**Purpose:**

This study investigates the effectiveness of through‐slice gradient optimization in dynamic slice‐wise B_0_ shimming of the cervico‐thoracic spinal cord to enhance signal recovery in gradient‐echo (GRE) EPI sequences commonly used in functional MRI studies.

**Methods:**

Six volunteers underwent MRI acquisitions with dynamic shim updating (DSU) using a custom‐built 15‐channel AC/DC coil at 3 T. A magnetization‐prepared rapid gradient echo was acquired to segment the spine and to provide a clear image of the anatomical region of interest in the figures. GRE B_0_ field maps were used to measure field homogeneity before and after shimming; the pre‐shimming field map was used for optimization. Shimmed fields were dynamically applied to GRE–echo planar imaging acquisitions simulating functional MRI acquisitions under two shimming conditions: DSU with and without through‐slice gradient consideration.

**Results:**

DSU with through‐slice gradient optimization increased the temporal signal‐to‐noise ratio at the T2 vertebral level by 201% compared with volume‐wise shim and by 28% compared with DSU without through‐slice. The residual geometric distortions were similar between DSU with and without through‐slice gradient optimization. A high signal loss penalty parameter was effective in simulations for reducing through‐slice gradient‐induced signal loss but led to instability and reduced image quality in actual acquisitions due to excessive in‐plane B_0_ inhomogeneities.

**Conclusion:**

Introducing a carefully balanced through‐slice gradient parameter in slice‐wise shimming substantially improves signal recovery in axial GRE images of the spinal cord, without compromising in‐plane homogeneity. This effective approach can advance spinal cord functional MRI applications at high field strengths.

## 1 INTRODUCTION

### 1.1 Challenges of spinal cord imaging

MRI of the spinal cord presents unique challenges due to the spinal cord's anatomical location limiting efficient receive coil coverage, its elongated size, and susceptibility‐induced magnetic field distortions. The proximity of the spinal cord to air–tissue interfaces, such as those between the lungs and the surrounding tissues, creates significant dynamic B_0_ inhomogeneities that vary with respiration.^1^, ^2^ Coupled with the spatially periodic B_0_ variations caused by the bone–tissue interface of vertebral discs,^3^, ^4^ it is difficult to achieve accurate volume shimming. These inhomogeneities can lead to signal loss and image distortions in gradient‐echo (GRE) echo planar imaging (EPI). Furthermore, the small cross‐section size of the spinal cord requires high in‐plane resolution for axial acquisitions,^5^, ^6^ which implies relatively thick slices (typically, 3–5 mm) to maintain an adequate signal‐to‐noise ratio (SNR). However, thick slices can introduce through‐slice dephasing, resulting in signal loss in GRE imaging.

### 1.2 Advanced B_0_ shimming solutions

Various approaches have been proposed to correct B_0_ inhomogeneities in the spinal cord. One approach tailors the region of interest (ROI) to match the spinal cord's curvature, improving field homogeneity when combined with scanner spherical harmonics^7^ or external shim‐only coils.^8^ This customization allows for more precise field correction than the scanner's rectangular shim box.

Dynamic slice‐wise shimming^9^ (DSU) refines this process by enabling slice‐specific shim adjustments, often incorporating the customized ROI to optimize local field corrections. This method has been effectively implemented in the spinal cord using scanner spherical harmonics^10^, ^11^, ^12^, ^13^ and multicoil systems.^14^ Among these, integrated parallel reception, excitation, and shimming coils (iPRES^15^, ^16^, ^17^; AC/DC^18^, ^19^) represent specialized multicoil designs that enhance DSU by providing highly localized field corrections. These approaches reduce distortions and improve signal recovery in the targeted region. To mitigate time‐varying distortions, real‐time shimming extends these methods by monitoring the breathing cycle and dynamically correcting for respiration‐induced field variations.^14^, ^20^, ^21^

Although these shim optimizations penalize ΔB_0_ in each voxel in a least‐squares sense, they do not explicitly penalize through‐slice gradients. Hence, despite their advantages, dynamic and real‐time shimming techniques primarily focus on in‐plane B_0_ corrections and have limited impact on through‐slice gradients, leaving signal loss in thick‐slice GRE spinal cord imaging unaddressed.

### 1.3 Optimizing through‐slice gradient for signal recovery

To address the issue of through‐slice gradients in two‐dimensional spinal cord imaging, researchers have developed slice‐specific z‐shimming techniques, which involve applying tailored z‐gradients, updated on a slice‐by‐slice basis, to reduce dephasing.^22^, ^23^, ^24^ The process typically begins with acquiring EPI calibration scans where the z‐gradient varies (e.g., 10 steps) across multiple volumes. These multiple acquisitions are then used to identify the z‐gradient that provides the highest signal for each slice. Tsivaka et al. extended slice‐specific z‐shimming to xyz‐shimming,^25^ where the x, y, and z gradients are all optimized to further enhance image quality.

The slice‐specific z‐shimming technique offers several advantages, notably increasing signal and temporal SNR (tSNR). Finsterbusch demonstrated that while a linear fit of the field effectively minimizes dephasing, it does not fully maximize signal recovery.^26^ This method avoids this problem by optimizing signal intensity rather than addressing through‐slice dephasing. Despite its advantages, this technique is limited, as it does not account for the effect of gradients on in‐plane B_0_ homogeneity, which remains a key challenge for reducing geometric distortions. Additionally, this method can only address static field variations, as acquiring multiple EPI scans at different time points during the breathing cycle for each tested gradient would introduce significant time constraints. These time limitations also restrict slice‐specific z‐shimming to a low number of degrees of freedom. As seen with xyz‐shimming,^25^ expanding the degrees of freedom increases calibration complexity and lengthens scan times. For example, a calibration scan for xyz‐gradients took 5 min and 33 s to acquire 125 volumes for only five gradient values—a significant drawback in clinical applications in which time efficiency is crucial.

Recent advancements in optimization algorithms have introduced a through‐slice gradient parameter into the mathematical mean squared error optimization process.^27^ This technique is synergistic with multicoil systems, which offer greater degrees of freedom than the scanner's linear gradients for high‐spatial‐order B_0_ shimming. Shim coils can also be used for dynamic shim updating, enabling real‐time adjustments to compensate for field variations. Because the optimization is field‐based, this approach can effectively correct dynamic field inhomogeneities induced by breathing. Although Willey et al. demonstrated the potential of this method in the brain,^27^ it is anticipated that it could be even more beneficial for the spinal cord, where through‐slice dephasing is very prevalent due to the use of thick axial slices and the presence of strong B_0_ inhomogeneities in GRE‐EPIs, especially in the lower cervical and thoracic cord.

The current study focuses on the following objectives:

1. Implement DSU with the additional through‐slice gradient parameter inside Shimming Toolbox^28^;
2. Assess the benefits of this new shim optimization scheme in the human cervical and upper thoracic spinal cord at 3 T using a 15‐channel AC/DC coil; and
3. Investigate the trade‐off between signal recovery and geometric distortions when DSU is used.

## 2 METHODS

### 2.1 Theory

In this section, we describe the algorithm used to compute the shimming coefficients, along with the regularization term designed to account for through‐slice dephasing. The signal recovery implementation in this work is based on a mean squared error (MSE) optimization.

The optimization function for MSE is defined as follows:

(1) $$\mathrm{mi}n_{I}\;\frac{1}{N}\sum_{a=1}^{N}{\left(\Delta B_{0,\text{shimmed}}\left(I\right)\right)}^{2}$$

where $\Delta B_{0,\text{shimmed}}$ represents the shimmed field as follows:

(2) $$\Delta B_{0,\text{shimmed}}\left(I\right)=\Delta B_{0,\text{baseline}}+\sum_{c=1}^{n}I_{c}\cdot \Delta B_{0,c}$$

where Δ*B*_0,baseline_ is the baseline field; Δ*B*_0,c_ is the coil profile of channel *c*; *N* is the number of voxels; *I*_c_ is the current in channel *c*; and *n* is the number of channels in the coil.

In most implementations of dynamic shimming, where this optimization is applied slice by slice, the through‐slice gradient is not explicitly compensated for. Although some optimization methods^28^, ^29^, ^30^, ^31^, ^32^ account for the adjacent slices (or an additional slice in through‐slice direction^10^) when performing slice‐wise shimming, adjacent slices are not a good surrogate of the true dephasing within a given slice.

To address this issue, Willey et al.^27^ modeled the signal loss *L* explicitly as shown in Eq. (3), assuming a rectangular slice profile, uniform spin density across the slice, and a linear field variation through the slice:

(3) $$L=1-\left|\mathrm{sinc}\left(\frac{\phi }{2}\right)\right|$$

where Φ is the phase dispersion:

(4) $$\phi =\gamma \frac{\partial B_{0}}{\partial z}\;\Delta z\cdot \mathrm{TE}$$

where γ is the gyromagnetic ratio; $\frac{\partial B_{0}}{\partial z}$ is the through‐slice gradient; ∆z is the slice thickness; and TE is the echo time.

In Eqs. (3) and (4), the through‐slice gradient is the only parameter that can be adjusted through shimming. Therefore, Eq. (1) was modified to incorporate signal recovery optimization, as follows:

(5) $$\mathrm{mi}n_{I}\;\frac{1}{N}\sum_{a=1}^{N}\left({\left(\Delta B_{0,\text{shimmed}}\left(I\right)\right)}^{2}+w{\left(\frac{\partial B_{0,\text{shimmed}}\left(I\right)}{\partial z}\right)}^{2}\right)$$

where *w* is a weighting parameter that balances the signal recovery objective with the standard MSE optimization.

### 2.2 Participants

Six healthy volunteers (3 males, 3 females; mean age 24.3; standard deviation 0.47) underwent MRI scans on a Siemens 3T Prisma‐fit scanner. All procedures were approved by an institutional review board and followed ethical guidelines. Participants were selected to ensure a balanced representation of both sexes, with no specific age or health status criteria.

### 2.3 Experiment

**FIGURE 1:**
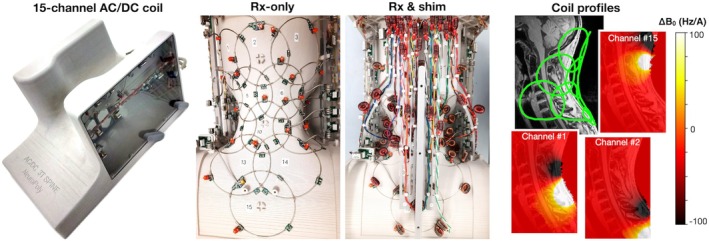
Bottom view of the 15‐channel AC/DC coil before (receive array; Rx‐only) and after (AC/DC; Rx and shims) adding the shimming capabilities. The AC/DC coil loops' positions are superposed on the T_1_‐weighted image with an example for three different channels' generated fields (coil profiles).

Participants were scanned using a custom‐built 15‐channel AC/DC coil^18^ (Figure 1). In the AC/DC approach, B_0_ shim capability is introduced into the loops of a radiofrequency (RF) receive array using high‐impedance chokes to create a DC path through the loop.^19^ The coil's DC component was driven by open‐source shim amplifiers.^31^ Currents were restricted to 2.5 A per channel and 25 A for all 15 channels.

#### 2.3.1 Baseline acquisition

After the localizer, a shim box was automatically fitted to the ROI, and an adjustment volume was performed with the scanner's zeroth to second‐order spherical harmonic shims.

Using this new shim, a baseline GRE‐EPI (axial, 1 × 1 × 5 mm, TE = 32 ms, repetition time [TR] = 2070 ms, flip angle [FA] = 70°, partial Fourier = ⅞, field of view [FOV] = 96 × 50 × 125 mm^3^, no GRAPPA, lipid saturation, 10 volumes) was acquired in both anterior–posterior (AP) and posterior–anterior (PA) phase‐encoding directions to assess geometric distortions induced by the B_0_ field. A PA sequence with 60 volumes was also collected to calculate the tSNR.

A magnetization‐prepared rapid gradient echo (MPRAGE) (1 × 1 × 1 mm^3^, TE = 3.72 ms, TR = 2000 ms, FA = 9°, FOV = 192 × 260 × 320 mm^3^, GRAPPA = 3) scan was obtained for segmentation and visualization of the spinal cord. Finally, two GRE B_0_ field maps were acquired. The first field map, used for the optimization (axial, 2.8 × 2.8 × 2 mm^3^, TE = [2.68 ms, 4.92 ms], TR = 705 ms, FA = 65°, FOV = 180 × 180 × 200 mm^3^, no GRAPPA), consisted of 100 slices to achieve enhanced resolution in the slice direction. The second field map (axial, 1.4 × 1.4 × 5 mm^3^, TE = [2.68 ms, 4.92 ms], TR = 20 ms, FA = 13°, FOV = 180 × 180 × 125 mm^3^, no GRAPPA) was acquired to match the slice coverage of the EPIs, enabling subsequent acquisition of the shimmed field.

The additional shimming steps took approximately 16 min: 10 min to set up the AC/DC coil (e.g., bring the DC cable into the room, connect it to the coil, switch on the shim amplifier, connect it to the laptop running *Shimming Toolbox*), 3 min and 28 s for the MPRAGE, 45 s for the field map, and 2 min for the segmentation and optimization. This time could be shortened using the localizer as the segmented image instead of the MPRAGE. The additional time required for this advanced shimming setup is further discussed in Section 4.3.

#### 2.3.2 Optimization

**FIGURE 2:**
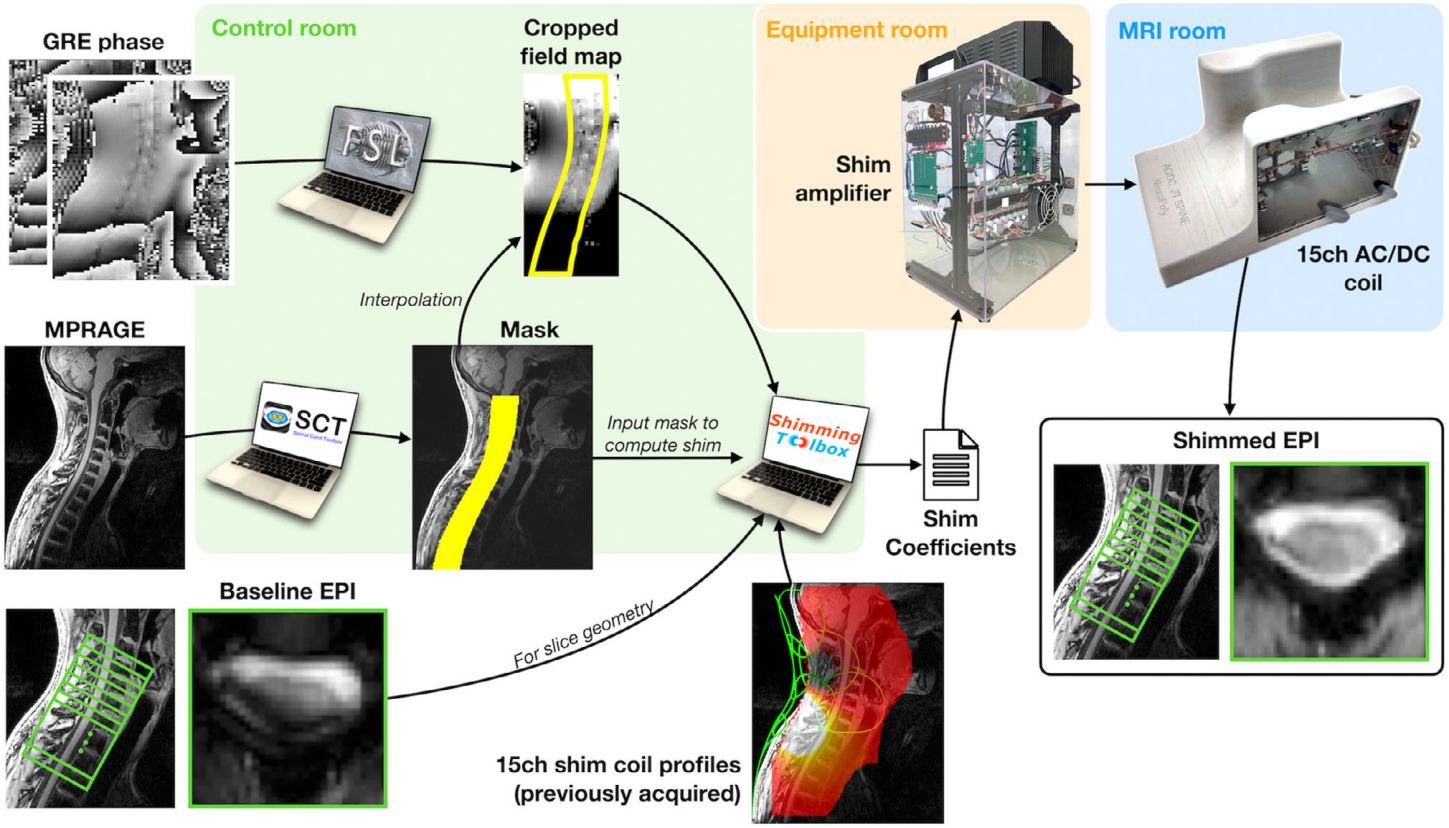
Pipeline of the experiments. Gradient‐recalled echo (GRE) phases from the field mapping sequence are passed along with the mask generated by *SCT* ^33^ to the *Shimming Toolbox* ^28^ (FSL's prelude^34^), which returns a field map cropped to fit the mask. The field map, the mask, the echo‐planar imaging (EPI), and the coil profiles for the 15‐channel AC/DC coil are sent to the *Shimming Toolbox*, which returns the shimming coefficient for the required optimization. The coefficients are then set on the shim amplifiers, which control the coil's currents during the experiment to acquire a shimmed EPI. MPRAGE, magnetization‐prepared rapid gradient echo.

Figure 2 illustrates the optimization pipeline (described subsequently).

The baseline acquisition DICOM files were sorted and converted to NIfTI format using *Shimming Toolbox*.^28^ Subsequently, the spinal cord on the MPRAGE was segmented using *Spinal Cord Toolbox* ^33^ (SCT v6.3) with the *sct_deepseg* command^35^ and the contrast agnostic model.^36^ With this segmentation, SCT generated two cylindrical masks centered on the spinal cord. The field map mask had a diameter of 40 mm, whereas the optimization mask had a diameter of 25 mm. The field map mask was larger to ensure accurate resampling due to the differing orientations of the MPRAGE and EPI slices.

Next, the field map was computed using *Shimming Toolbox* and the 100‐slice GRE acquisition. Using this field map, the 15‐channel AC/DC coil currents were optimized for each EPI slice and two signal loss parameters: 0 (regular slice‐wise shim, no consideration for the through‐slice gradient) and 0.01. The optimization was conducted to minimize the mean squared error of the B_0_ field using a least‐squared optimizer. One of the 6 subjects was evaluated with two additional optimization parameters (0.0001 and 1) to assess the effect of the signal loss parameter. The value of 0.01 was based on simulations with various parameter values on field maps acquired before the first experiment. The Python tool *scipy.optimize.minimize()* ^37^ was used to solve the least square equation to find optimal shim currents subject to current constraints.

#### 2.3.3 Shimming acquisitions

Each optimization acquisition followed a standardized procedure. Initially, the optimized currents were uploaded to the shim amplifiers. Subsequently, all baseline EPIs (AP, PA, tSNR) were reacquired. The EPI pulse sequence was modified to generate a transistor‐transistor logic (TTL) trigger before each RF pulse. The shim amplifiers captured these triggers and adjusted the AC/DC coil currents for the subsequent slice.

Because the field optimization was confined to a small mask, the field outside could interfere with fat saturation. To avoid this issue, an additional TTL trigger was inserted 2 ms before every fat saturation pulse to allow time for the microcontroller to zero out the currents. Following the EPI acquisitions, the baseline B_0_ field map with the same slices as the EPIs was acquired. The GRE sequence to map the B_0_ field was also modified to send a TTL trigger before each new slice.

## 3 RESULTS

**FIGURE 3:**
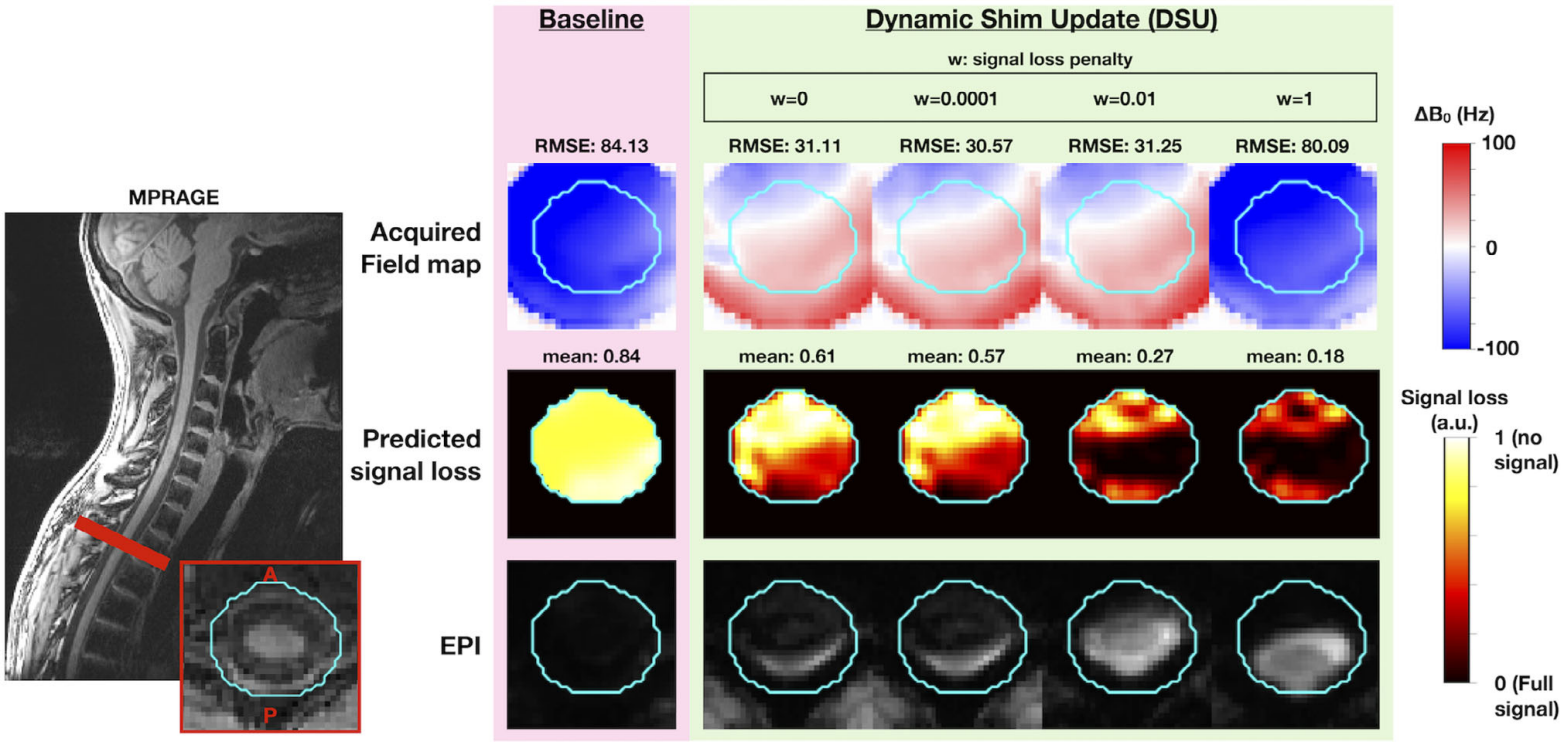
Magnetization‐prepared rapid gradient echo (MPRAGE), acquired field map, predicted signal loss maps, and echo‐planar images (EPIs) for one slice of the same subject for the baseline and for different signal loss penalty parameters (*w* = [0, 0.0001, 0.01, 1]) from Eq. (5). The signal loss maps were computed using Eq. (3). The light blue circle is the contour of the region of interest used for the shimming optimization. The contour was initially in the MPRAGE reference frame and was resampled in the EPI's reference frame. All optimizations apart from baseline were dynamically shim updated (DSU) with the 15‐channel coil's channels. RMSE, root mean square error.

Figure 3 illustrates the trade‐off between signal loss and B_0_ slice homogeneity. An MPRAGE image of the same slice imaged by the EPI serves as a reference, depicting the spinal cord's geometry without signal loss or geometric distortions. The figure demonstrates a sharp and consistent decrease in B_0_ root mean square error (RMSE) when using DSU with a signal penalty parameter (*w*) of 0.01 or lower. In contrast, a higher value (*w* = 1) results in a B_0_ field RMSE similar to the baseline. This is evident in the EPI images, where the spinal cord in the final column (*w* = 1) appears distorted in the posterior direction due to B_0_ inhomogeneities.

Regarding signal loss, even DSU, which does not account for signal recovery (*w* = 0), shows improvement in the signal loss map, as the optimizer includes contributions from the slice above and below the target slice. However, the signal loss significantly decreases with *w* = 0.01 and 1, resulting in much higher signal quality in the EPI images. Notably, there is a clear correspondence between the signal loss maps and the EPIs. For instance, the *w* = 0.01 signal loss map shows a drop in signal at the top of the ROI, which is also reflected in the corresponding EPI image.

It is important to note that with a *w* of 1, the required shimming currents were substantially higher (approximately 15 A per slice, compared with less than 5 A for other optimizations), and the image quality across slices was inconsistent.

**FIGURE 4:**
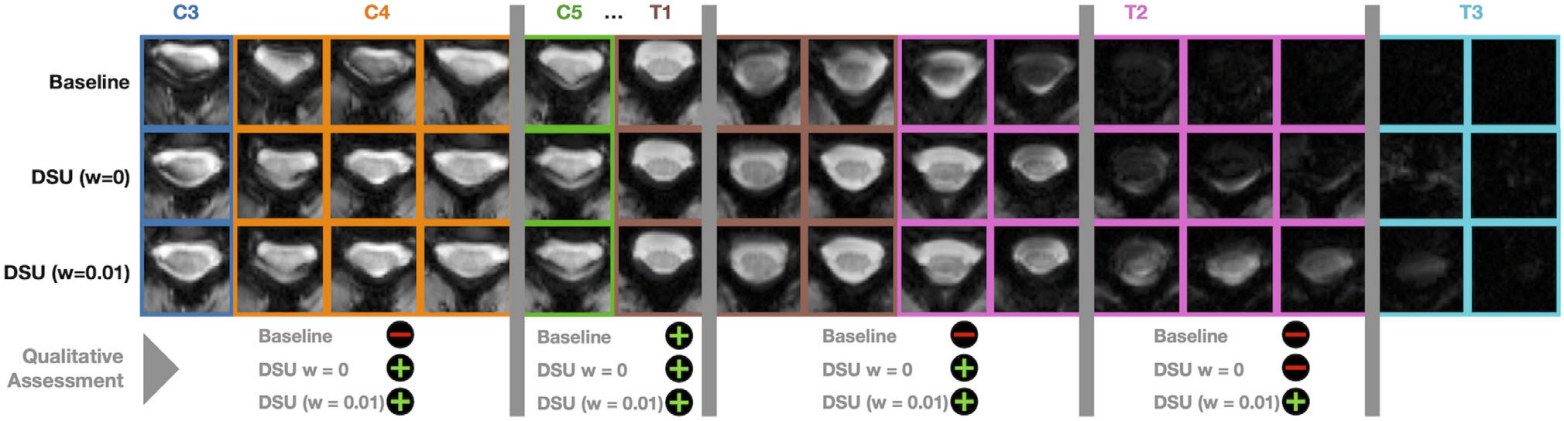
Twenty‐five axial slices covering C3 to T3 vertebral levels, cropped around the spinal cord, for three scenarios: baseline, dynamic shim updating (DSU) without signal loss penalty (*w* = 0), and DSU with signal loss penalty (*w* = 0.01). All optimizations apart from baseline were dynamically shim updated with the 15‐channel coil's channels. The image scaling is the same across the three scenarios.

Figure 4 presents all EPI slices for Subject 6 across three acquisitions: baseline (scanner shim), dynamic shim update (DSU) with no signal loss penalty (optimizing only for in‐plane B_0_ homogeneity), and DSU with a 0.01 signal loss penalty (optimizing for both in‐plane B_0_ homogeneity and through‐slice gradient). Image quality in the baseline acquisition is notably worse in the C3 and C4 slices. From C5 to C7, the baseline offers comparable geometric distortions and signal drop‐offs to the other two shimmed acquisitions. Below T1, a clear signal drop‐off is observed in the baseline acquisition compared with the DSU conditions. The DSU without signal recovery optimization (*w* = 0) begins to diverge from the DSU with signal recovery (*w* = 0.01) at T2, and no acquisitions provide a visible spinal cord at the T3 level.

**FIGURE 5:**
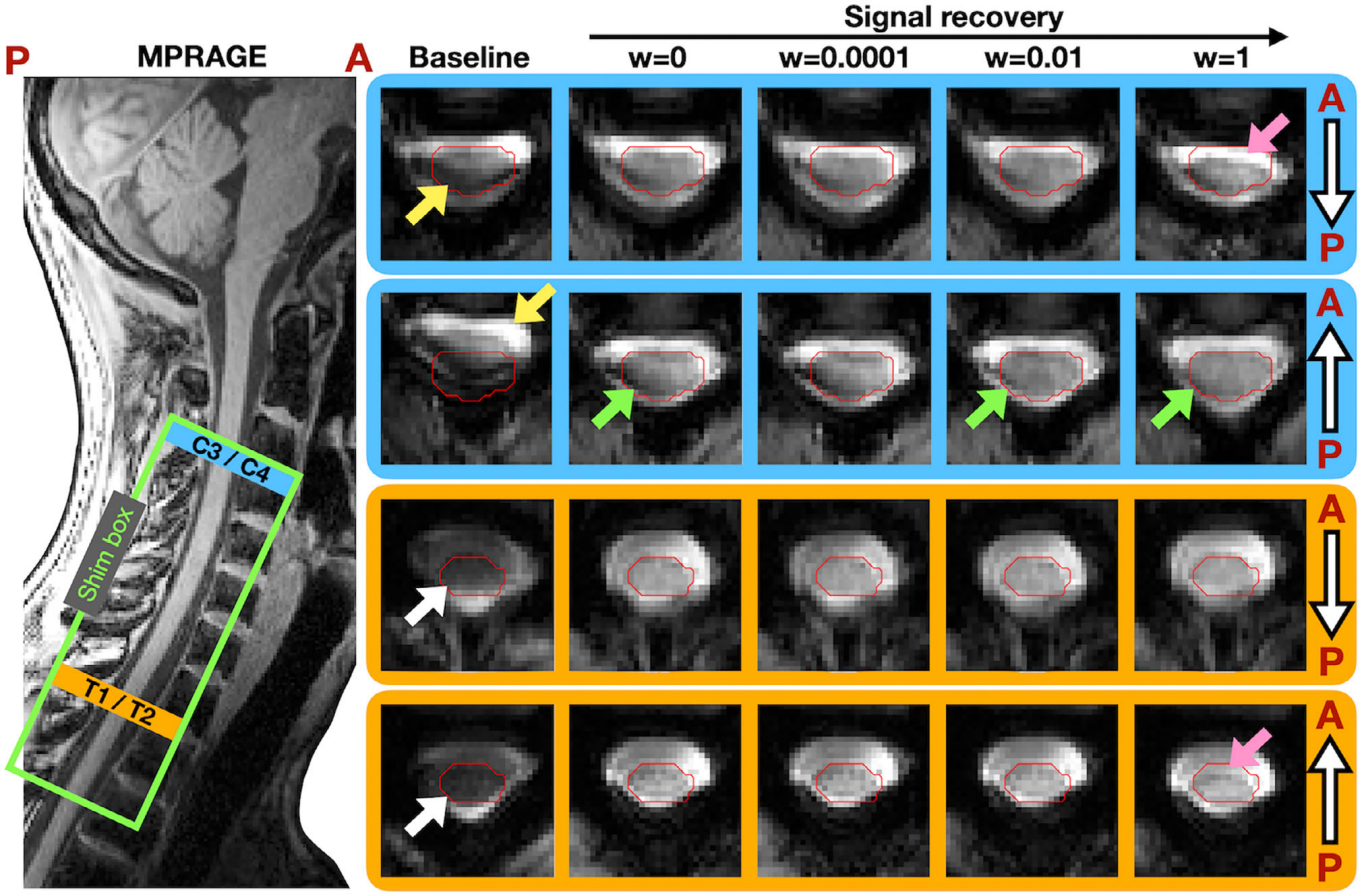
Echo‐planar images (EPIs) for two slices were acquired in anterior (A)–posterior (P) and PA phase‐encoding directions. The anatomical locations of the shown slices are indicated on the magnetization‐prepared rapid gradient echo (MPRAGE). The red spinal cord contour is based on the MPRAGE image and has been resampled in the EPI space. The yellow arrows show the geometric distortions in the baseline acquisition; the green arrows show the signal loss in the C3/C4 slice; the pink arrows show the geometric distortions in the w = 1 acquisitions; and the white arrows show the signal loss in the baseline acquisition. All optimizations apart from baseline were dynamically shim updated with the 15‐channel coil's channels.

Figure 5 presents two EPI slices from the same subject, for AP and PA phase‐encoding directions. As *w* increases, the distortions become more pronounced. This is expected, as higher *w* values shift the optimization's focus toward minimizing the through‐slice gradient at the expense of in‐plane B_0_ homogeneity, thereby exacerbating geometric distortions. Conversely, signal loss is also reduced by the increase of *w*. The baseline volume‐wise shim acquisition shows even greater geometric distortions in slices where the initial B_0_ homogeneities were poorly corrected by the scanner's zeroth to second‐order spherical harmonic shim. Likewise, the presence of a relatively high z‐gradient in the lower cord causes strong signal loss in the baseline shim, which is well recovered by dynamic shimming. The C3/C4 slice exhibits geometric distortions that are more pronounced than the T1/T2 slice, despite the latter typically being more challenging due to its proximity to the lungs. This artifact will be further discussed in the next section.

**FIGURE 6:**
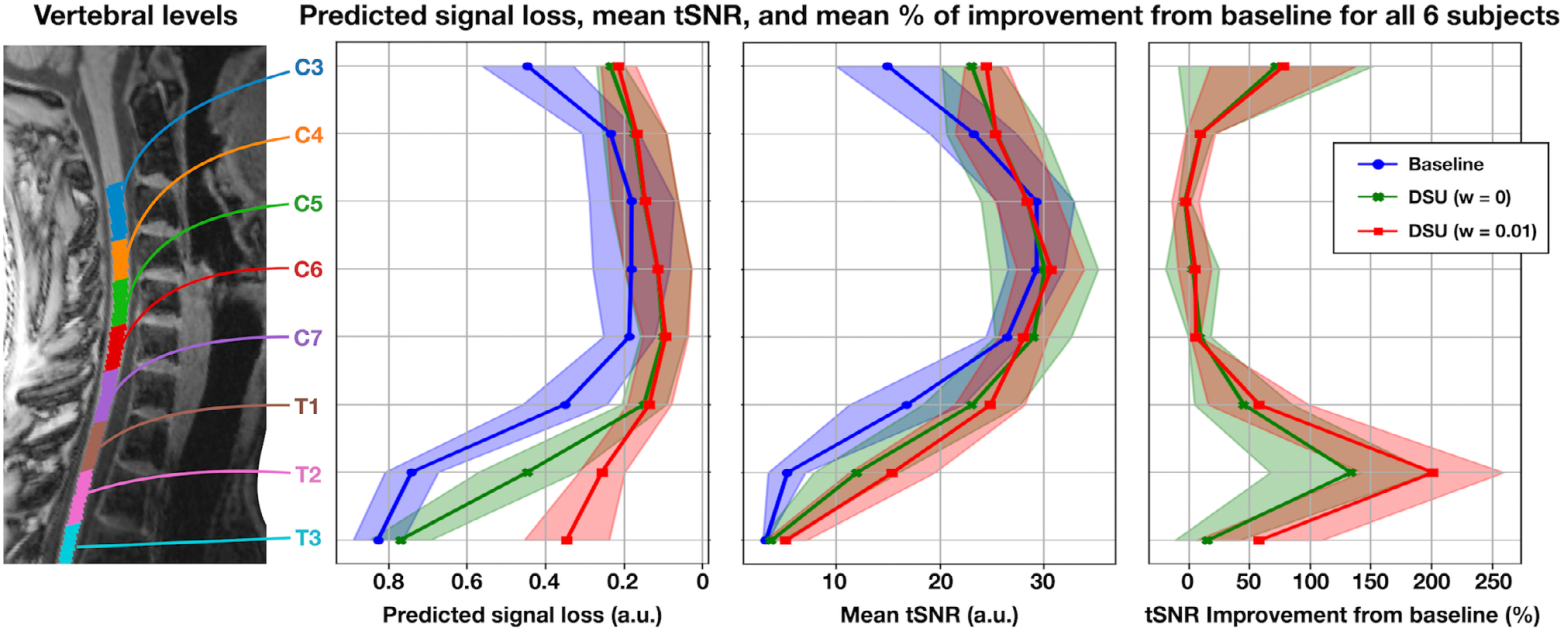
Predicted signal loss, mean temporal signal‐to‐noise ratio (tSNR), and tSNR improvement from baseline for all 6 subjects with the standard deviation. Each of the 25 slices was distributed to vertebral levels using *SCT*'s segmentation (*left*). The values on the plots represent the average of every slice attributed to the vertebral level. Improvement from baseline represents the individual improvement from each subject relative to their baseline acquisition. All optimizations apart from baseline were dynamically shim updated (DSU) with the 15‐channel coil's channels.

**FIGURE 7:**
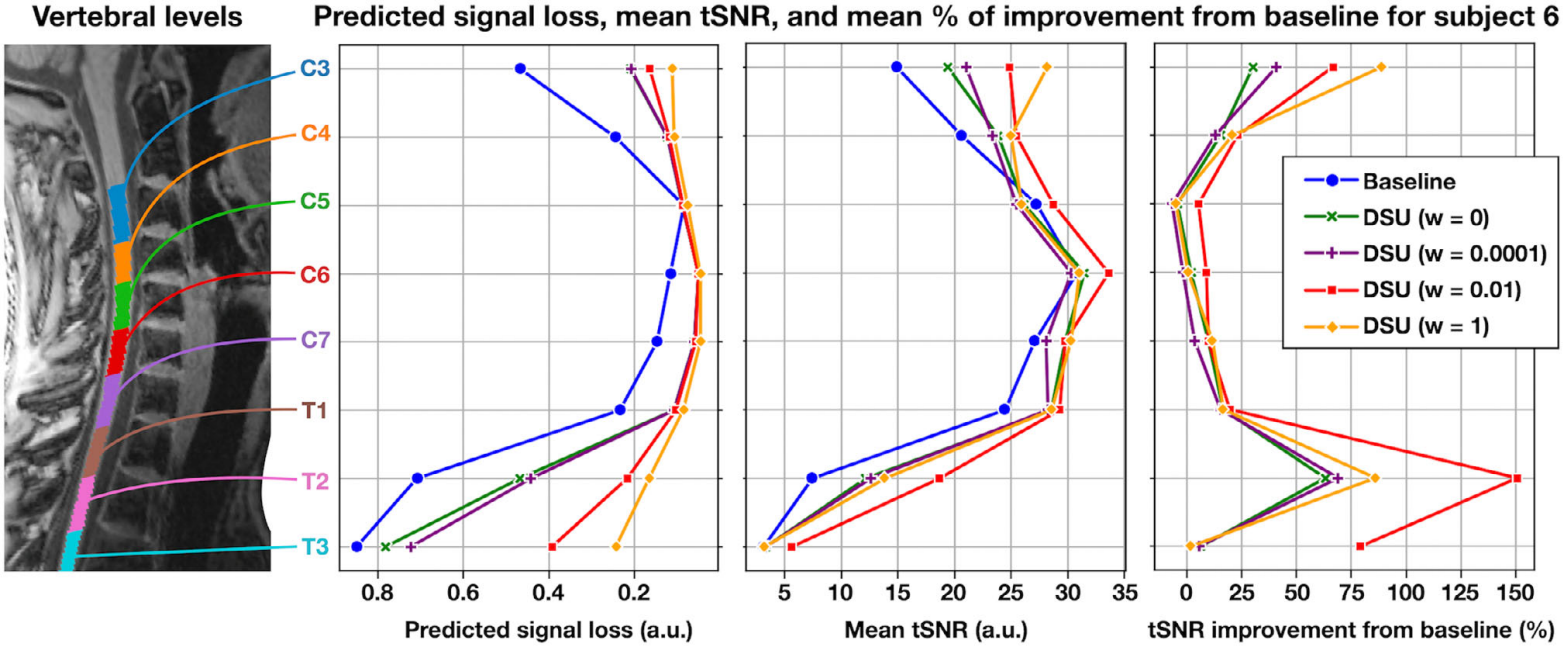
Predicted signal loss, mean temporal signal‐to‐noise ratio (tSNR), and tSNR improvement from baseline for Subject 6 as the optimization parameter is varied (baseline, *w* = [0, 0.0001, 0.01, 1]). Each of the 25 slices was distributed to vertebral levels using *SCT*'s segmentation on the left. The values on the plots represent the average of every slice attributed to the vertebral level. All optimizations apart from baseline were dynamically shim updated (DSU) with the 15‐channel coil's channels.

Figures 6 and 7 illustrate the effect of the signal loss penalty on predicted signal loss across different vertebral levels and its correlation with the acquired tSNR and tSNR improvements from the baseline acquisition. Predicted signal loss was computed with *Shimming Toolbox* using Eq. (3); mean tSNR was calculated from the 60 EPI volumes using the code available on GitHub; and tSNR improvement was the average improvement in each vertebral level for each subject. Signal loss is most pronounced at the T2 and T3 vertebral levels, whereas it is minimal from C4 to T1, with a slight increase at the edge of the volume of interest in C3. The tSNR follows a similar pattern to the signal loss.

For different shimming methods, a clear trend emerges between the predicted signal loss and the acquired tSNR from C3 to T1. However, at T2 and T3, although DSU with a signal loss penalty parameter of 0.01 yields better tSNR, the improvement does not fully reflect the predicted signal recovery. With a higher parameter value (*w* = 1), the lower slices proved even more challenging to shim than expected, resulting in an overall tSNR lower than that achieved with the lower signal recovery parameter (*w* = 0.01).

Overall, the greatest signal improvements from signal recovery optimization are observed at T2, where the initial signal loss was substantial, and in C3, where a notable drop‐off in signal was observed in the baseline acquisition, with 201% and 78% average improvements, respectively.

**FIGURE 8:**
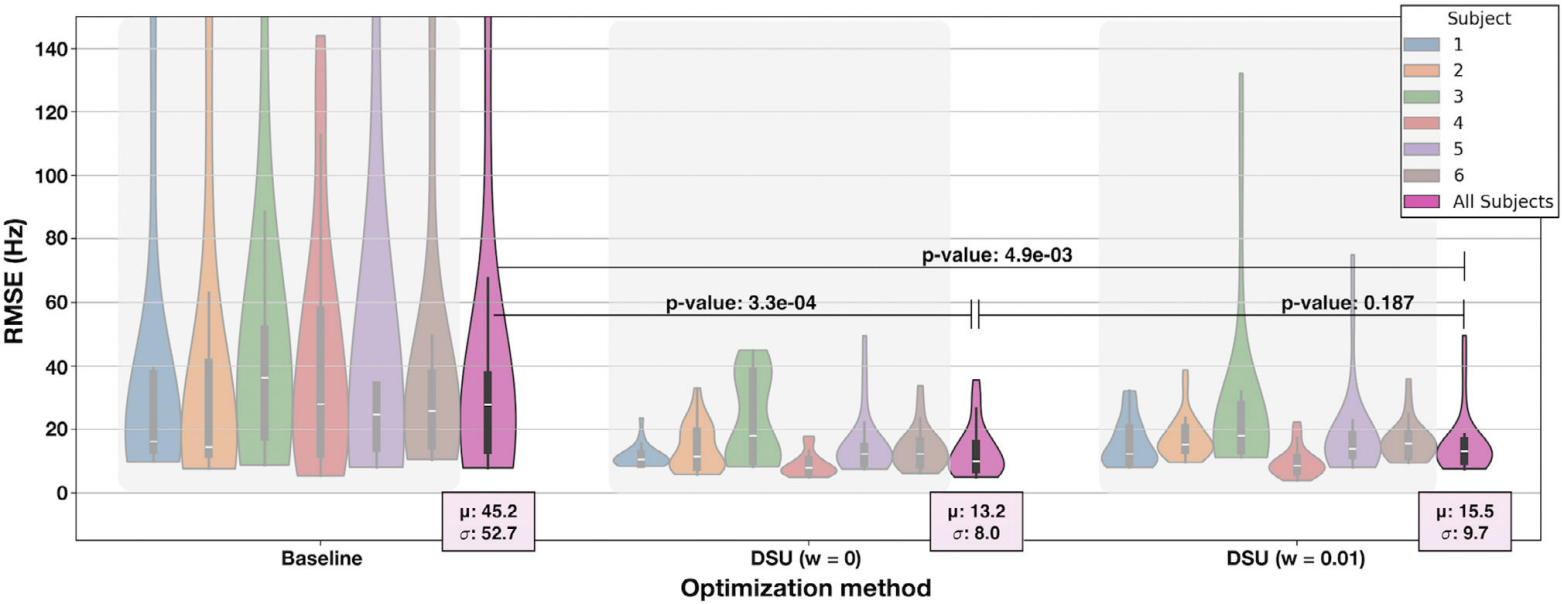
Violin plots of the mean in‐plane B_0_ root mean square error (RMSE) for every slice of every shim for all 6 subjects. The dynamic shim updates are optimized with different *w* values from Eq. (5). The mean and standard deviation of the distribution were computed for each optimization of each subject. *p*‐Values were calculated using the Mann–Whitney *U* test comparing the combination of all subjects into a single distribution (all subjects). All optimizations apart from baseline were dynamically shim updated with the 15‐channel coil's channels.

**FIGURE 9:**
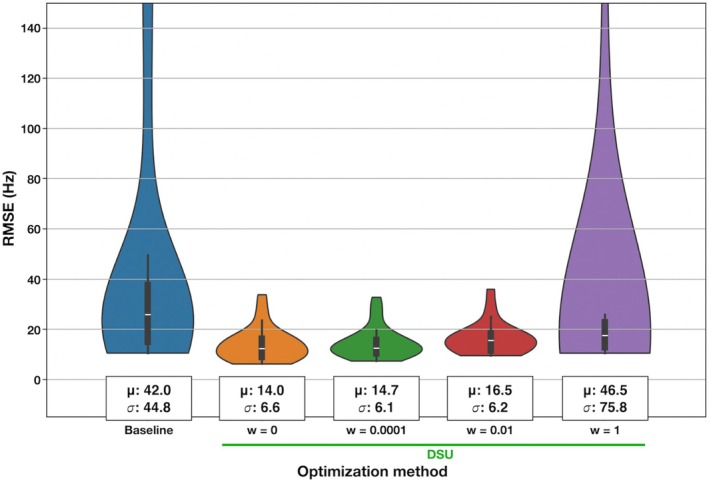
Violin plots of the mean in‐plane B_0_ root mean square error (RMSE) for every slice for Subject 6. The mean and standard deviation of the distribution were computed for each optimization. As the signal loss penalty (*w*) from Eq. (5) increases, the optimization favors signal recovery over in‐plane B_0_ homogeneity. All optimizations apart from baseline were dynamically shim updated (DSU) with the 15‐channel coil's channels.

Figures 8 and 9 display the in‐plane B_0_ RMSE distribution across all axial slices, with Figure 8 showing all 6 subjects and Figure 9 focusing on Subject 6, who was tested with two additional *w* parameters. The use of DSU led to a mean reduction in RMSE over all 6 subjects of 71% when optimizing only for in‐plane B_0_ homogeneity (*w* = 0) and a 66% reduction when jointly optimizing in‐plane B_0_ homogeneity and the through‐slice gradient (*w* = 0.01). The higher RMSE values, particularly noticeable in Subjects 2 and 4 during signal recovery optimization (*w* = 0.01), are due to lower slices in T3, where signal loss is severe and the spine is not visible in the EPIs, even after shimming. This causes the optimizer to overcompensate for the through‐slice gradient, leading to a higher in‐plane B_0_ RMSE. When comparing the different optimizations' axial B_0_ RMSE with the baseline using a Mann–Whitney *U* test, both showed a significant difference (*w* = 0: p_val_ = 3.3e‐04 < 0.05; *w* = 1: *p*_val_ = 4.9e‐03 < 0.05). No difference was found when comparing them with each other (p_val_ = 0.187 ≥ 0.05). In Figure 9, for Subject 6, B_0_ RMSE is similar across *w* values of 0, 0.0001, and 0.01. However, at the other extreme, using a *w* of 1 results in a worse overall RMSE than the baseline acquisition.

## 4 DISCUSSION

This study evaluated dynamic (slice‐wise) shimming with an additional through‐slice gradient term that accounts for signal recovery, applied to the cervicothoracic spinal cord (C3 to T3). We compared this optimization scheme against conventional volume‐wise shim, and dynamic shimming without this additional through‐slice gradient term. Overall, the through‐slice gradient was beneficial in the reduction of signal loss caused by intravoxel dephasing. In the following discussion, we address the specific benefits of the additional through‐slice gradient optimization term in the spinal cord, discuss the choice of the *w* parameter, and evaluate the existing limitations of the methods to propose future steps.

### 4.1 Benefits and limitations of through‐slice gradient optimization

#### 4.1.1 Signal recovery

Dynamic shimming with through‐slice gradient optimization (*w* = 0.01) proved to be highly effective in the upper thoracic region (Figures 4, 6, and 7), with tSNR improvements over conventional volume‐wise shimming of 58%, 201%, and 58% for T1, T2, and T3 vertebral levels, respectively. In comparison, dynamic shimming without through‐slice gradient optimization (*w* = 0) showed more modest tSNR gains of 46%, 134%, and 16%. This is reflected in Figure 3, where the through‐slice penalty term greatly improves the signal at the T2 vertebral level while maintaining similar in‐plane B_0_ characteristics. In the cervical region, both dynamic shimming methods showed similar global tSNR results; however, when looking closely at the images, we notice a reduction of signal loss in some subjects and imaging slices. For example, in Figure 5 we notice a region of signal loss caused by intravoxel dephasing (green arrow, *w* = 0), which is partially recovered with the through‐slice gradient term (*w* = 0.01 and *w* = 1). The moderate improvement of dynamic shimming at C3–C7 levels could be because the baseline volume‐wise shimming is already providing a reasonably good homogeneity; therefore, the effective gain of dynamic shimming and/or of the additional through‐slice gradient term is minimal. A few reasons for a reasonably good volume shim include: (i) the B_0_ field in the C3–C7 region being more homogenous than in the thoracic region (T1–T3) before the volume shim; (ii) C3–C7 benefitting from good SNR (owing to the excellent coil sensitivity profile in this region) for both the EPI and the B_0_ field map data; and (iii) the spinal cord (at least in that young subject) being further from intervertebral discs, which does not cause the small spatially scale inhomogeneities that are typically seen at the vicinity of the posterior tip of the disc.

However, Figure 4 shows that no shimming optimization achieved sufficient image quality in the lower T2 and T3 regions. This is likely due to the highly temporally varying B_0_ field in this region, resulting from patient respiration and the relatively low RF receive coil sensitivity. This is discussed later in Section 4.3.

#### 4.1.2 In‐plane distortions

A limitation of adding a through‐slice gradient term is that the in‐plane homogeneity (measured with B_0_ RMSE) could suffer, leading to more in‐plane geometric distortions. When optimizing only for in‐plane B_0_ homogeneity in a slice‐wise fashion (*w* = 0), B_0_ RMSE was reduced by 71% compared with conventional volume‐wise shimming. Conversely, when incorporating the through‐slice gradient in the optimization (*w* = 0.01), B_0_ RMSE was reduced by 66%, only 5% less compared with the “classical” dynamic shimming scenario. This 5% difference was statistically not significant (Mann–Whitney U test; *p* = 0.187). Looking at Figure 5, we indeed observe a similar spinal cord shape between the *w* = 0 and the *w* = 0.01 cases. Therefore, introducing a signal loss penalty of 0.01 effectively enhanced signal recovery, particularly in the lower slices (T1 to T3), without compromising B_0_ homogeneity compared with standard in‐plane optimization.

Interestingly, when looking at the baseline (volume‐wise) scenario from Figure 5, we notice more in‐plane distortions at level C3/C4 (blue color) versus T1/T2 (orange). This is counterintuitive, as we generally expect more B_0_ field inhomogeneity in the upper thoracic cord compared with the upper cervical cord. This unexpected result may be related to how the vendor's volume shim operates. Specifically, optimal shim coefficients are calculated based on voxels inside the green box shown in Figure 5; however, given the relatively spatially smooth variation of the B_0_ field, voxels inside the shim box have more contribution than those at the edge of the shim box (such as those at the C3/C4 level), because those at the edge do not benefit from the contribution from neighboring voxels outside the shim box (in this case, right above the C3/C4 level). The volume shim was a critical starting point in this experiment, since the slice‐wise shimming only relies on the external AC/DC coil. In future studies, the scanner gradients and the external AC/DC coils should be optimized jointly for slice‐wise shimming, hopefully addressing this issue.

### 4.2 Adjusting the signal loss penalty term

A limitation of adding a through‐slice gradient term is that the in‐plane homogeneity could suffer. Therefore, we need to find a fine balance between the homogenization of B_0_ and the reduction of the through‐slice gradient. This balance is set by the term *w* in Eq. (5). The effect of the signal loss penalty *w* was further investigated in 1 of the 6 subjects by repeating the acquisitions for two additional *w* parameters: 0.0001 and 1.

A small penalty (*w* = 0.0001) produced results similar to regular B_0_ optimization (*w* = 0) in both in‐plane B_0_ RMSE and signal loss, suggesting that such a small penalty does not offer any noticeable improvements.

At the other extreme, using a *w* of 1 for through‐slice gradient optimization showed only marginal improvements in the predicted signal loss compared with a penalty of 0.01, while significantly increasing B_0_ RMSE. This optimization resulted in a worse overall field than the baseline acquisition, as illustrated in Figure 9. Moreover, the improved predicted signal loss did not yield better tSNR in the lower part of the FOV (Figure 7). One likely explanation is that the required coil currents were more than 3 times higher on average (15 A/slice vs. 4 A/slice for the lower *w* parameter). Higher currents often lead to more unstable acquisitions and less reliable results, as any errors in the generated field scale proportionally with the current. Additionally, because the coil currents are set to zero during the fat saturation pulse that precedes each slice acquisition, the larger current transitions might induce eddy currents. These steeper transitions could also cause current instability at the start of each acquisition, leading to an unstable field. Another reason why the higher signal loss penalty (*w* = 1) did not yield better tSNR than the lower penalty (*w* = 0.01) was that the predicted signal loss improvements were marginal and not concentrated in the spinal region where tSNR is measured. The shimming mask used was slightly larger than the spinal cord (25 mm diameter), meaning that improvements can occur outside the region of interest.

Ultimately, a signal loss penalty of 0.01 proved to be the optimal value of *w* in our sampling set, striking the best balance between minimizing geometric distortions and maximizing signal recovery. Whether this quasi‐optimal value holds in other applications (e.g., other body parts, voxel resolution, different AC/DC coil shape) remains to be investigated. Preliminary results performed in the brain, using another coil (32‐channel) and voxel resolution (axial 1 × 1 × 1 mm^3^ and axial 2 × 2 × 2 mm^3^), also suggested *w* = 0.01 to work well for that application.^38^ From these experiments, the *w* parameter appears to be resolution‐agnostic. A trade‐off value of 0.01 maintains in‐plane homogeneity comparable to that of a trade‐off of 0 while reducing the through‐slice gradient. Adjusting *w* might become necessary when introducing a second weighting factor to minimize in‐plane gradients. In this case, additional analysis would be required to determine the optimal trade‐off parameters, which would likely depend on the resolution, as the impact of in‐plane gradients on signal loss relative to through‐slice gradients varies with voxel size.

### 4.3 Limitations and future steps

A notable finding observed in some subjects was that signal recovery optimization occasionally resulted in worse signal loss in small specific regions of the spine, even though the overall signal loss within the ROI was lower than traditional B_0_ optimization. This issue arises because the binary mask used for the ROI is larger than the spinal cord itself, leading to a focus on overall signal loss without consideration for where the loss occurs within the mask. A soft mask could address this while maintaining a more flexible ROI that includes a small region outside the spinal cord (to avoid distortions near the spine). This soft mask would assign a value of 1 to voxels within the spinal cord and gradually reduce the value from 1 to 0 using a Gaussian curve for voxels outside the cord.

To further improve image quality, future signal‐recovery optimization could account for the subject's breathing cycle, which induces field variations as described in previous studies.^1^, ^2^ Breathing causes a B_0_ offset and generates a through‐slice gradient, as the f_0_ offset varies depending on the location along the spine. Incorporating this into the signal‐recovery optimization could help mitigate breathing‐induced through‐slice gradients.

Another limitation of this study is the design of the shim coil. Our AC/DC coil was optimized for the cervical spine, with a higher density of receive and shim loops in that region, but it only features one loop near the T2 and T3 vertebral levels, limiting the degrees of freedom for shimming and SNR in this area. This limitation is especially problematic when current in the more distant loops is restricted. Designing a new coil optimized for the thoracic spine could be crucial for further improving GRE‐EPI images in this region.

The proposed advanced shimming protocol required an additional 16 min, including the hardware setup, acquiring the T_1_‐weighted anatomical scan and field map, and running optimization. However, this time could be shortened using the localizer as the segmented image instead of the MPRAGE, reducing it to about 12 min. If an AC/DC coil with the shimming setup is already preinstalled, the 10‐min setup time could be reduced to 2 min.

## 5 CONCLUSIONS

In this study, we evaluated the effectiveness of dynamic slice‐wise shimming and signal‐recovery optimization in reducing signal loss and improving image quality in the cervicothoracic spine. Results demonstrated that incorporating a signal loss penalty (*w* = 0.01) provided the optimal balance between minimizing geometric distortions and enhancing signal recovery, particularly in the upper thoracic region, where through‐slice gradient minimization proved essential. Although limitations in coil design affected performance at the thoracic levels, future improvements, such as using a soft mask and accounting for breathing‐induced field variations, could further enhance the technique's potential. Given its quick and efficient optimization process, this method holds promise for improving functional MRI studies and two‐dimensional GRE anatomic images in the spine.

## FUNDING INFORMATION

Funded by the Canada Research Chair in Quantitative Magnetic Resonance Imaging [CRC‐2020‐00179], the Canadian Institute of Health Research [PJT‐190258], the Canada Foundation for Innovation [32454, 34824], the Fonds de Recherche du Québec–Santé [322736, 324636], the Fonds de Recherche du Québec–Nature et technologies [329439], the Natural Sciences and Engineering Research Council of Canada [RGPIN‐2019‐07244], the Canada First Research Excellence Fund (IVADO and TransMedTech), the Courtois NeuroMod project, the Quebec BioImaging Network [5886, 35450], Mila–Tech Transfer Funding Program, and the National Institutes of Health NIBIB [R01 EB028797, and U24 EB028984].

## Data Availability

All scripts used for this paper are available on this GitHub repository: https://github.com/shimming‐toolbox/spinalcord‐signal‐recovery/releases/tag/v1.0. All data acquired for this paper are available on OSF at this link: https://osf.io/rs6tv/.
